# microRNA-based biomarker for dementia

**DOI:** 10.18632/aging.101868

**Published:** 2019-03-12

**Authors:** Kensuke Toyama, Masaki Mogi, Philip S. Tsao

**Affiliations:** 1 Department of Pharmacology, Ehime University Graduate School of Medicine, Ehime, Japan; 2 Division of Cardiovascular Medicine, Stanford University School of Medicine, Stanford, CA 94305, USA; 3 VA Palo Alto Health Care System, Palo Alto, CA 94304, USA

**Keywords:** microRNAs, exosome, dementia, blood-brain barriers, miR-501-3p

Dementia is one of the biggest public health issues for aging individuals worldwide. There is concern that the number of dementia patients worldwide will increase approximately 3-fold, from 46.8 million to 113.5 million, by 2050. Alzheimer’s disease represents more than 60% of dementia cases. However, two-thirds of Alzheimer’s disease patients also display signs of cerebral vascular injury. When combined, the proportion of patients with dementia related to vascular disorders (including pure vascular dementia) is nearly the same as that of Alzheimer's disease, suggesting that vascular factors play an important role in cognitive pathology. This phenomenon of so-called “vascular cognitive impairment” invites therapeutic strategies that focus on the blood vessels. Indeed, numerous studies have indicated that vascular risk factors are associated with various forms of dementia including Alzheimer's disease. Observational studies have revealed that hypertension, dyslipidemia, middle-aged obesity, type 2 diabetes and smoking are high risk for the incidence of dementia, and a recent meta-analysis suggests that interventions addressing vascular risk factors, especially hypertension and diabetes, lower dementia incidence [1].

The blood-brain barrier (BBB) protects the brain from harmful substances and provides homeostasis for optimal neuronal function, with vascular endothelial cells serving as key components in its gateway role. The finding that BBB breakdown is involved in the initiation and progression of dementia has been recently highlighted [2,3]. The primary methods for evaluation of BBB disruption in the clinical setting include imaging, such as magnetic resonance imaging and single photon emission computed tomography, but these constitute a considerable burden in time and cost. Although several markers, such as S100A12 [4], have been reported as promising biomarkers to evaluate BBB status, none have yet achieved clinical utility. If BBB status could be evaluated easily though clinical examination, it would become a powerful tool for exploring new therapeutic strategies, not only for cognitive decline but also for other neurological disorders associated with BBB disruption, and for accelerating future studies.

MicroRNAs (miRs) are small, single-stranded RNAs with a length of ≈22 nucleotides, that typically repress messenger RNA (mRNA) expression by binding to the 3′-untranslated region, and which influence many major (patho)physiological processes. The study of miRs currently represents one of the most active areas of research in genomics. Considerable work to-date has shown that miRs are key regulators of numerous complex genetic programs relevant to cardiovascular pathology and vascular disease, as well as nervous system disorders. We recently found that "miR-501-3p" plays an important role in BBB disruption though the regulation of tight junctions, and that it is one of the key miRs in the development of vascular cognitive decline [5].

**Figure 1:**
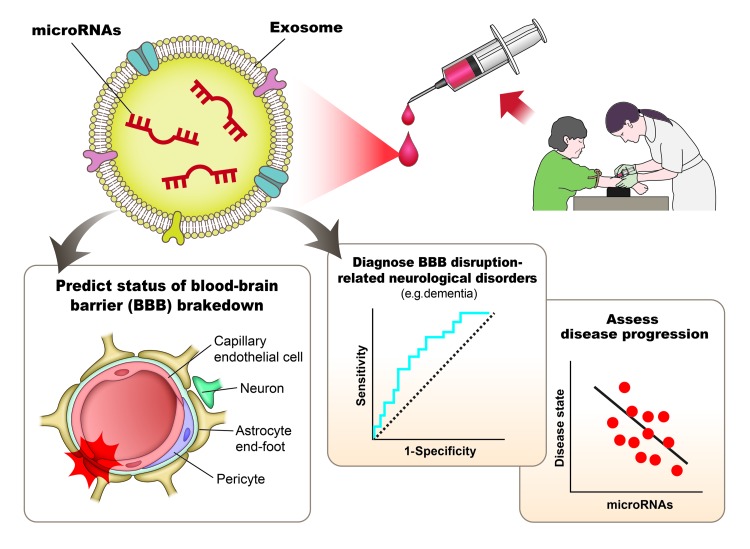
Concept of microRNA-based diagnostic assessment in cognitive decline and/or in predicting blood-brain barrier dysfunction status.

Exosomes are extracellular vesicles secreted from most cell types, 30 nm to 200 nm, which contain both mRNAs and miRs, and travel to distant tissues to directly influence various aspects of cell behavior, often more rapidly than traditionally recognized methods of local gene-expression control [6,7]. Several miRs have been reported to be promising biomarkers in such conditions as cancer and cardiovascular disorders, and have shown high stability in body fluids including plasma as exosome-miRs, suggesting their usefulness in characterizing disease state. For example, circulating miR-208 has been proposed as a biomarker of myocardial infarct state [8]. Whether the blood-borne exosome-miR-501-3p may represent a biological marker for evaluating BBB dysfunction, or for diagnosing BBB disruption-related neurological disorders such as cognitive decline and for assessing disease progression requires further investigation. It is essential to establish whether evaluation of BBB breakdown or dysfunction through the measurement of the blood exosome-miRs is viable in the clinical setting. If so, this may assist with the development of optimal treatments for central nervous system disease with BBB dysfunction including dementia (Figure 1).
